# The Immunopathology of Preeclampsia

**DOI:** 10.3390/biomedicines14071591

**Published:** 2026-07-16

**Authors:** Jenny Valentina Garmendia, Humberto Azpurua, Alexis Hipólito García, Juan Bautista De Sanctis

**Affiliations:** 1 Institute of Molecular and Translational Medicine, Faculty of Medicine and Dentistry, Palacky University, Hnevotinska 1333/5, 779 00 Olomouc, Czech Republic; 2 Department of Obstetrics and Gynaecology, Hospital Universitario General de Catalunya, 08195 Barcelona, Spain; humberto.azpurua@gmail.com; 3 Institute of Immunology “Nicolás Enrique Bianco”, Faculty of Medicine, Universidad Central de Venezuela, Caracas 1050, Venezuela; alexisgarcia27@gmail.com

**Keywords:** preeclampsia, innate immunity, autoantibodies, NK cells, Th1, Th17, Treg

## Abstract

Preeclampsia (PE) is a hypertensive disorder of pregnancy characterized by target organ damage, affecting approximately 5% of pregnancies. Complex neuroendocrine alterations, vascular imbalances, excessive oxidative stress, environmental factors, and inappropriate immune responses drive the pathology of this condition. Inadequate remodeling of the uterine spiral arteries serves as a fundamental marker of the disease. PE is heavily mediated by an inflammatory cascade involving complement system activation, decreased tolerogenicity of natural killer (NK) cells, M1 macrophage polarization, and dendritic cell alterations. Furthermore, the disease is characterized by a shift toward Th1, Th17, and Th22 cell populations, alongside a decrease in Th2 and regulatory T (Treg) lymphocytes, significantly increasing the risk of maternal autoimmunity. The disorder also disrupts angiogenesis, alters specialized pro-resolving lipid mediators, and impairs responses to infections. Although advancements in immunological treatments have been made, many therapeutic approaches remain under active investigation.

## 1. Introduction

Preeclampsia (PE) is a pregnancy disorder characterized by new-onset hypertension (greater than 140/90 mmHg) and end-organ damage, typically arising after the 20th week of gestation and affecting approximately 5% of pregnancies [1,2,3]. A primary indicator of PE is the improper remodeling (transforms narrow, high-resistance uterine vessels into wide, low-resistance conduits) of the uterine spiral arteries during early pregnancy [1,2,3,4]. This inadequate process triggers increased blood pressure and multi-organ dysfunction, which can progress to severe forms such as Hemolysis, Elevated Liver enzymes and Low Platelets (HELLP) syndrome and eclampsia [2,3,4].

PE is driven by a complex interplay of neuroendocrine changes, vascular imbalances, oxidative stress, and genetic and environmental factors that collectively result in a dysregulated immune response [2,3]. The condition typically presents two distinct phenotypes. Early-onset PE (before 34 weeks) is driven by superficial trophoblastic invasion, altered uteroplacental perfusion, and early immune alterations, often presenting with fetal growth restriction. Conversely, late-onset PE (after 34 weeks) constitutes 80% of cases, involves late syncytiotrophoblast alterations, and is closely associated with maternal cardiometabolic variables [1,2,3]. Early-onset preeclampsia is believed to have significant associations with uteroplacental insufficiency, likely resulting from inadequate placentation. In contrast, late-onset preeclampsia is thought to be more closely linked to maternal risk factors, including elevated body mass index, diabetes mellitus, and nulliparity. Subclusters of early and late PE have been proposed to enhance the understanding of this condition. Currently, PE is widely recognized as a severe inflammatory disorder that is characterized by significant dysfunction of the maternal immune system. This delineation of the two stages is crucial for further exploration and comprehension of the disease’s complexities [3,4,5].

The objective of the present review is to highlight alterations in the immune response associated with PE and other cellular processes. Additionally, the review will provide a comprehensive overview of the impact of infectious diseases. Finally, it will discuss the treatment strategies currently being investigated and proposed.

## 2. Methodology

A comprehensive literature search was conducted across electronic databases, including PubMed/MEDLINE, Scopus, and Web of Science, for peer-reviewed articles published through June 2026. The search strategy focused on identifying high-quality evidence regarding preeclampsia, immune response, oxygen and nitrogen radicals, cell death, angiogenesis, cytokines, immunogenetics, extracellular vesicles, inflammation resolution, and treatment. The search included original articles, meta-analyses and reviews on immunology and preeclampsia, focusing on the following on complement, leukocytes (neutrophils, eosinophils, mast cells, macrophages, dendritic cells, myeloid-derived suppressor cells, NK cells, NKT cells, T gamma-delta lymphocytes, T and B lymphocytes, Th1, Th2), cytokines, leptin, angiogenesis, galectins, HLA, KIR, immune checkpoints, leukotrienes, thromboxanes, prostaglandins, resolvins, microARNs, extracellular vesicles, and free radicals (including oxidative stress). Also, infectious diseases and treatment.

## 3. Innate Immunity in Preeclampsia

### 3.1. Complement

PE is associated with heightened activation of both the alternative and terminal complement pathways, as well as diminished regulatory mechanisms. Complement over-activation is commonly observed in severe preeclampsia subtypes, especially in HELLP syndrome [6,7].

Complement split products, such as C4d and C5b-9, accumulate significantly in placental tissue, thereby compromising the interface at which maternal blood interacts with fetal cells [6,7]. As critically validated markers, elevated plasma concentrations of the complement components Bb, C5a, and the terminal complex C5b-9, also referred to as the membrane attack complex, indicate increased inflammation and placental injury [5,6]. Furthermore, heightened deposition of C5b-9 in the placenta triggers the release of soluble fms-like tyrosine kinase-1 (sFlt-1), which directly contributes to endothelial dysfunction [5,6,7].

Elevated levels of Factor Bb during the early stages of pregnancy have been identified as a significant biomarker associated with an increased risk of developing [6,7]. Furthermore, substantial urinary excretion of the terminal complement complex, C5b-9, is associated with disease severity [6,7,8]. Consequently, overactivation of the complement system leads to severe placental alterations, characterized by notable increases in C3a, C5a, and C5a-C9 activation products, which are frequently observed in HELLP syndrome [5,6,7,8].

Certain genetic variations in complement regulatory proteins have been identified as factors that may increase women’s susceptibility to severe, early-onset PE and associated conditions [7,8]. Nonetheless, this area of research remains in its developmental stages, necessitating well-designed clinical studies to establish definitive findings.

Given the critical role of complement regulation in preeclampsia, various therapeutic interventions have been evaluated and are discussed in the therapy section at the conclusion of the manuscript.

### 3.2. Neutrophils and Eosinophils

In the context of PE, neutrophils are integral to the systemic inflammation and vascular damage that define the condition. Increased neutrophil counts, along with a heightened Neutrophil-to-Lymphocyte Ratio (NLR), are widely acknowledged as accessible and reliable early indicators of both the risk and severity of PE [9]. Neutrophils are recruited to the placenta through various chemokines, including IL-8/CXCL8, pro-inflammatory cytokines such as TNF-α, and chemotactic lipids [10]. These biochemical signals are predominantly released by the decidua, endometrial epithelial cells, and placental trophoblasts in response to factors such as inflammation, placental debris, or bacterial invasion, including conditions like chorioamnionitis.

Neutrophils produce reactive oxygen species (ROS) that damage the vascular endothelium, promoting hypertension and end-organ injury. Activated neutrophils also interact with decidual endothelial cells, increasing resistance in the placental bed [11,12], and interfere with crucial placental angiogenesis [12]. Furthermore, they disrupt the balance of angiogenic factors by favoring sFlt-1 production, thereby promoting decidual vascular injury [13,14]. Additionally, neutrophil extracellular traps (NETs) are strongly associated with systemic inflammation and inefficient placental perfusion characteristic of PE [15,16].

According to Kong et al. [17], an analysis of first-trimester immune markers indicates that neutrophils may be significantly associated with hypertension in pregnancy. In contrast, monocytes, platelets, and lymphocytes were associated with peripheral inflammation and an increased risk of developing PE. Therefore, the role of neutrophils as key players in hypertension and preeclampsia requires further detailed studies.

PE is characterized by lower eosinophil counts than in normotensive pregnancies; early eosinopenia may serve as a diagnostic marker of inflammation and immune activation, although it has not been validated [18]. The decreased number of circulating eosinophils appears to be associated with elevated levels of the chemokine CCL8 in PE [19]. The role of these cells in pregnancy and related pathologies is not well understood, underscoring the need for further research.

### 3.3. Mast Cells

Mast cells normally contribute to placental formation; however, in PE, their abnormal activation exacerbates inflammation and alters vascular tone [20]. Mast cells serve as the principal storage sites for histamine, a potent vasoconstrictor that affects placental vasculature. In PE, placental histamine concentrations are generally elevated [20]. Activated mast cells release proteolytic enzymes, including tryptase and chymase [21]. Chymase is particularly notable for its role in converting angiotensin I into angiotensin II, a process that contributes to maternal hypertension [21]. Furthermore, mast cells produce growth factors, such as vascular endothelial growth factor (VEGF), to promote the growth of healthy blood vessels. However, in PE, this process is disrupted [22,23,24,25]. Increased mast cell activity in this condition is associated with a higher rate of extravascular fibrosis and reduced vascularization.

Broekhuizen et al. [26] reported that these innate alterations occur early in early-onset PE, but not in late-onset PE, implying distinct pathophysiological phases. More research should focus on the roles of these cells and their pharmacological modulation in PE.

The presence of other conditions characterized by systemic dysregulation of mast cells, such as Mast Cell Activation Syndrome (MCAS), has been associated with an elevated risk of pregnancy complications [27]. These complications include PE, hyperemesis gravidarum, and premature delivery [27]. Additionally, pregnant individuals with atopic disorders or severe asthma, in which mast cells play a crucial role in the immune response, may experience an increased incidence of PE [28]. It has been suggested that non-adherence to asthma treatment during the first trimester and low vitamin D levels prior to pregnancy may contribute to this association [29]. Further clinical studies are needed to confirm this hypothesis.

### 3.4. Macrophages

During normal pregnancy, tissue-resident macrophages are crucial for remodeling uterine spiral arteries [30,31]. In PE, macrophage dysfunction leads to insufficient placental development [31]. In preeclamptic women, during the second and third trimesters, inflammatory M1 macrophages dominate the placenta and affect local endothelial cells [31]. This increase in the M1/M2 ratio promotes chronic inflammation by increasing the secretion of TNF-α, IL-1β, IL-6, and IL-18, which are associated with reduced blood flow and endothelial damage [31]. In parallel, IL-10 production is reduced, thereby directly impairing trophoblast invasion and contributing to systemic hypertension and proteinuria [31,32,33].

Decidual macrophages play a crucial role in the immunological processes involved in pregnancy. Their interaction with decidual NK cells within the tolerogenic decidual environment is essential for maintaining normal gestation. These cellular components are considered integral to the pathophysiology of PE and represent a viable target for therapeutic intervention.

### 3.5. Dendritic Cells (DCs)

In PE, dendritic cells (DC) exhibit a pro-inflammatory bias that disrupts maternal-fetal immune tolerance, impairing placental development and causing hypertension and organ damage [34,35]. During the first trimester of pregnancy, decidual cells that constitute the uterine lining secrete chemokines, notably CCL2 and CCL5, in response to inflammatory stimuli, such as Interleukin-1β [34,35]. This secretion facilitates the recruitment of dendritic cells and macrophages to the maternal-fetal interface. Upon their recruitment, these immune cells undergo alterations that can interfere with the normal formation of spiral arteries, subsequently leading to placental hypoxia and dysfunction [34,35]. This process further stimulates the release of pro-inflammatory factors [34,35].

In PE, an increased myeloid DC (mDC)- to-plasmacytoid DC (pDC) ratio promotes Th1 pro-inflammatory responses [35,36]. Abnormal DC activation in this disease triggers a cytokine imbalance favoring Th1 and Th17 profiles, worsening endothelial damage [13,35,36]. Furthermore, dysregulated DCs negatively influence local NK, NKT, and Tγδ cells [36,37], positioning them alongside macrophages as key initiators of PE [36].

Using flow cytometry, Sundarajan et al. [38] proposed that certain dendritic cell subsets, specifically CD1c+ and CD141+, exhibit distinct alterations that may be potential biomarkers for early-onset PE. The test is simple and non-invasive; however, it has not been validated on a large scale. Additionally, research is being conducted on regulatory dendritic cell therapies within preclinical models to prevent the onset of this condition.

### 3.6. Myeloid-Derived Suppressor Cells (MDSCs)

MDSCs exert crucial immunosuppressive effects that maintain fetal tolerance; they migrate to the placenta in a healthy pregnancy [39,40]. They act by suppressing T and NK cells in the decidua [41]. In PE, the number and function of granulocytic (G-MDSCs) and monocytic (M-MDSCs) subsets may be altered, and this imbalance drives inflammation and vascular dysfunction [32,33,34]. Low circulating MDSC counts in the blood and placenta correlate with disease severity, and their measurement could serve as an early disease marker [34]. PE is also associated with the inhibition of G-MDSCs and their effector enzyme, arginase I [42]. In contrast, the number of M-MDSCs and the expression of Tim-3 on these cells are increased [43]. This increase is accompanied by elevated levels of Galectin-9 in the placenta, which is the ligand for Tim-3 [43]. The role of these cells and their mediators is still under investigation.

### 3.7. Natural Killer (NK) Cells

NK cells play a critical role in local immune tolerance in normal pregnancy [36,44,45,46,47,48]. Uterine NK cells (CD3-/CD16dim/CD56bright) are vital for spiral artery remodeling and local immunotolerance [44,45,46,47,48]. In PE, NK cells are increased in the peripheral blood and decidual tissue (especially CD16+/CD56+) [36], and their function is also enhanced. In this disease, NK cells produce more IFN-γ and express more activating receptors, such as NKG2C and NKG2D [36,46,47,48,49,50,51,52]. Nevertheless, some authors have reported reduced decidual CD56+ NK cells, which may explain defective arterial remodeling [50,51,52,53].

The role of NK cells in the decidual environment is critical for the establishment of a healthy pregnancy, as previously reviewed [44]. However, even minor modifications to this environment can prompt a transition in these cells from a tolerogenic to a cytotoxic state [36,44]. There exist three distinct subpopulations of decidual NK (dNK) cells [44]. In the context of preeclampsia (PE), there is an observed shift among these subpopulations: a reduction in the tolerogenic dNK1 and an increase in the more cytotoxic dNK3, which is further associated with the secretion of inflammatory cytokines [36,44]. This alteration ultimately facilitates the migration and activation of macrophages and T cells, thereby intensifying the local inflammatory response.

Local suppressive compounds delivered with nanocarriers and cellular therapies have been proposed and partially studied, while significant research continues on the role of NK cell modulation in pregnancy-related complications [44,45].

### 3.8. NKT Cells

NKT cells, which serve as a link between innate and adaptive immunity, exhibit elevated levels in PE and contribute to systemic inflammation through a Th1-dominant profile [53]. The increased prevalence of these cells may impair decidual NK cell function, resulting in placental dysfunction. Furthermore, NKT cells can activate NK cells by producing IFN-γ, enhancing the inflammatory burden [36,52]. Pharmacological treatments for recurrent pregnancy loss and recurrent implantation failure can reduce the inflammatory effects of these cells [45], although specific mechanisms remain under investigation.

### 3.9. T Gamma-Delta Lymphocytes (Tγδ)

Tγδ cells recognize antigens independently of the major histocompatibility complex and are associated with a Th1 immune response [54,55,56]. These cells play a key role in the local tolerogenic response in normal pregnancy [45]. In pregnancies affected by PE, peripheral Tγδ cells show a significant increase in both abundance and function, including elevated secretion of perforin and INF-γ when compared to healthy pregnancies [57]. Nevertheless, more research is needed to understand the specific role of these cells in PE.

## 4. Adaptive Immunity in Preeclampsia

### 4.1. T Lymphocytes

T lymphocytes constitute a significant proportion of decidual mononuclear cells [58]. In PE, an imbalance in T lymphocyte subpopulations occurs, wherein effector cells increase while regulatory networks fail, rendering the fetus susceptible to maternal immune attack [45,59]. This cellular imbalance correlates strongly with elevated endoglin (a cytokine that neutralizes TGF-β) and widespread inflammation [59,60,61,62,63,64].

While a predominant Th2 immunotolerant profile characterizes a typical pregnancy, PE is distinguished by a severe pathophysiological shift toward a Th1-dominant response, characterized by the overproduction of pro-inflammatory cytokines such as IFN-γ, IL-2, and TNF-α [63,64].

Th17 cells drive robust inflammatory responses that play a key role in PE [58,59]. In PE, Th17-derived cytokines (IL-17, IL-21, and IL-22) are significantly elevated, thereby accelerating the inflammatory cascade of the disease [65,66,67,68,69,70]. Th17 levels correlate inversely with circulating regulatory T cells [71]. Th22 lymphocytes, which produce IL-22 but not IL-17, are likewise elevated in PE and involved in tissue remodeling and inflammatory response [59,69,70,72], though their precise pathogenic role remains unclear.

Tregs are essential for maintaining maternal-fetal immune tolerance [73]. In contrast to normal pregnancies [74,75], Tregs in PE are significantly decreased in number and function across both peripheral blood and decidual tissues [76,77,78,79]. The Treg/Th17 ratio is markedly reduced [11,43], making early quantification of Tregs a potential predictive biomarker for the disease [79,80]. Key Treg alterations in PE include reduced paternal/fetal antigen-specific Tregs, decreased CD4+CD25+FoxP3high (activated) Treg, and altered naïve/effector Treg balance [73]. In summary, in PE, maternal-fetal immune homeostasis breaks down, characterized by reductions in Tregs, tolerogenic dNK cells, and decidual macrophages, along with infiltration of effector T cells, resulting in chronic inflammation [44,45,73,79,80]. Advancements in pharmacological therapy remain necessary to mitigate cell migration and activation, which contribute to the onset of PE.

Even though quantifying Tregs and the Th17/Treg ratio is a simple flow cytometry test using a small amount of peripheral blood, it has not been validated as a routine test by the American or European societies.

### 4.2. B Lymphocytes and Preeclampsia

Assessment of B lymphocyte subsets—particularly CD19+CD5+ cells, IL-10–producing B cells, and B1 lymphocytes—represents a promising early risk marker for PE, although it has not been fully validated [11,81,82,83,84,85]. B cells play a critical role in PE pathogenesis by producing pathogenic autoantibodies, most notably those targeting the angiotensin II type I receptor, which directly elevate blood pressure and disrupt placental function [81,82,85]. Memory B cells play a crucial role in autoimmune diseases and likely also in PE [85]. Interestingly, few studies have examined the role of B cells in pregnant women with autoimmune diseases. Well-defined, validated autoimmune therapy may be important in several pregnancy complications. Further research is needed in this area.

## 5. Angiogenesis and Preeclampsia

Recent theories suggest that PE is a complex disease with two clinical presentations: the placental phenotype, characterized by superficial trophoblastic invasion and fetal growth restriction. In contrast, the maternal phenotype shows normal fetal growth but mild maternal inflammation due to placental oxidative stress and lesions [86,87].

The first stage of early gestation involves abnormal, asymptomatic placental invasion and differentiation. Normally, the embryo-derived cytotrophoblast penetrates the uterine wall and remodels the spiral arteries into wide, low-resistance vessels [88]. In PE, this invasion is incomplete and restricted to superficial decidual layers, leading to decreased uteroplacental perfusion and ischemia [88]. The second stage involves clinical signs of PE, in which chronic placental hypoperfusion leads to the abnormal release of bioactive factors into the maternal circulation [88,89,90,91]. These factors cause widespread endothelial dysfunction, vasospasm, reduced plasma volume, oxidative stress, and a hyperinflammatory state [88,89,90,91].

A key concern in pregnancy-related conditions like PE is vascular and endothelial dysfunction [2,90,91]. Placental ischemia releases anti-angiogenic and pro-inflammatory factors, leading to an imbalance in which anti-angiogenic factors exceed pro-angiogenic factors [88,90,91,92,93]. This imbalance drives endothelial dysfunction, arterial vasoconstriction, and hypertension [2,88,90,91,92,93].

Placental Growth Factor (PlGF) promotes trophoblast growth, while Vascular Endothelial Growth Factor (VEGF) supports endothelial health and vessel formation [88,92,93]. Both factors are pro-angiogenic and work together [88,92,93]. Soluble fms-like Tyrosine Kinase 1 (sFlt-1), a VEGF receptor variant, circulates in maternal blood and antagonizes both VEGF and PlGF, thereby hindering their action [88,92,93]. The placenta is the primary source of sFlt-1 during pregnancy, and its levels can be up to fivefold higher in patients with PE [2,88,92,93], leading to vasoconstriction and endothelial dysfunction. Decreased PlGF levels are associated with the onset of PE, and the sFlt-1-to-PlGF ratio is a reliable predictor of PE and its complications [2,94,95,96,97]. Additionally, soluble Endoglin (sEng), which regulates VEGF, TGF-β, and PlGF, is also elevated in PE patients [93,94]. Table 1 illustrates the clinical significance of the sFIT/PIGF ratio. These are clinically validated markers that are useful for making medical decisions in PE.

## 6. Galectins in Preeclampsia

**Figure 1:**
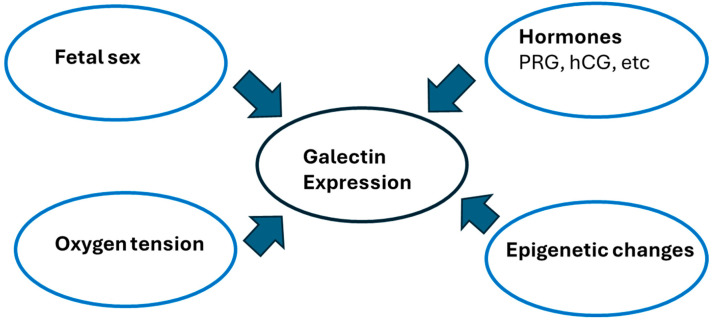
Events regulating galectin expression and the roles of different galectins in placentation. The hormones that regulate galectin expression are sex hormones (progesterone (PRG), 17-β estradiol, prolactin); however, in placentation, the key hormones are PRG and human chorionic gonadotropin (hCG). Hypoxia and epigenetic changes induced by environmental factors can also alter galectin expression.

Galectins are crucial carbohydrate-binding proteins for a healthy pregnancy, regulating embryonic implantation, maternal-fetal immune tolerance, and placental development [98,99]. In PE, their functions are impaired, and they may serve as clinical biomarkers [98,99]. Figure 1 illustrates the different events involved in galectin transcription and expression in pregnancy.

In normal pregnancy, galectin-1 is highly expressed in the endometrium and placenta, where it facilitates early trophoblast invasion, regulates maternal immune tolerance, and promotes the remodeling of spiral arteries [98,99]. Galectin-3, which is expressed across various trophoblast lineages, plays a critical role in cell migration, tissue remodeling, and angiogenesis [98,99]. Galectin-13, also known as Placental Protein 13 (PP13), is exclusively expressed in the placenta and is essential for the structural integrity necessary for the initial anchoring of the placenta to the uterus [99,100]. Galectin 14 is also associated with migration and invasion, and consequently, along with galectin 13, is critical for trophoblast survival [99,100].

**Figure 2:**
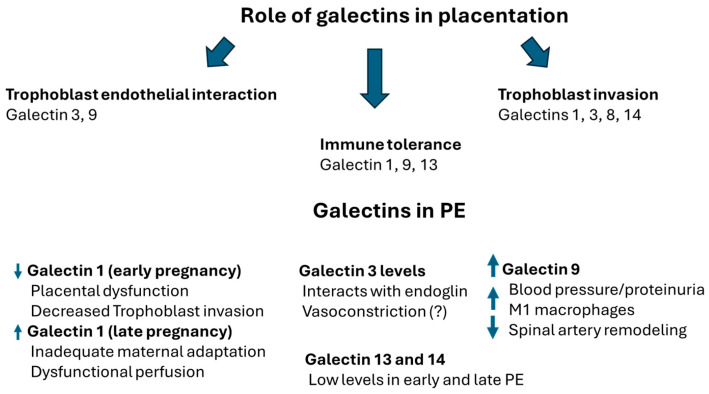
The Role of Galectins in Placentation and Preeclampsia. The influence of galectins on critical functions during placentation is depicted. Galectin expression is notably affected by various factors, as illustrated in Figure 1. Significant aspects of placentation include the involvement of galectins in trophoblast/endothelial interactions, the promotion of immune tolerance, and trophoblast invasion. The levels of galectins 1 and 9 have been identified as potential predictors of preeclampsia, as indicated in the figure. In contrast, galectins 13 and 14 consistently show low levels across both stages of preeclampsia. Furthermore, galectin 3 levels do not demonstrate specificity to the stage of preeclampsia.

In PE, impaired placental formation and immune tolerance lead to the release of anti-angiogenic factors such as sFlt-1 and to widespread inflammation [99,100]. Galectins play a key role in this process: elevated levels of galectin-7 disrupt normal placentation, increase anti-angiogenic factors, and contribute to hypertension [99,100]. Low serum galectin-3 levels are observed in impaired placentation in early-onset PE [101]. Galectin-1 low maternal serum levels in the first trimester are linked to poor placentation and adverse pregnancy outcomes [99,100,101]. Galectin 9 is also critical in PE, as increased production by trophoblasts is associated with a decreased remodeling of the uterine spiral arteries [102]. Figure 2 shows the different roles of specific galectins in placentation and their levels related to PE.

As the clinical markers for PE continue to be delineated, the evaluation of sFlt-1/PGIF has emerged as a validated method for characterizing the condition. Additionally, galectins 1, 3, and 9 may function as biomarkers within well-defined clinical cohorts. These biomarkers likely also serve as effective indicators of therapeutic efficacy during pregnancy.

## 7. Immunogenetics and Preeclampsia

### 7.1. The Role of Human Leukocyte Antigens (HLA) in Preeclampsia

HLA complexes, which differentiate self from non-self, are classified into class I (A, B, C; non-classical E, F, G) and class II [103,104]. Increased PE risk is associated with HLA-A matching, Class I matching, and combined Class I and Class II matching between mother and fetus [105]. In preeclamptic pregnancies, maternal-fetal HLA-C matching may be unexpectedly high [103,104,105], although some studies show no correlation [103]. In oocyte-donation pregnancies, greater fetal-maternal HLA class II mismatch increases the risk of PE [105,106,107]. The HLA-DPB1*04:01:01G allele is more frequent in severe PE/eclampsia [108]. Non-classical HLA-F and HLA-G molecules, which promote maternal immune tolerance, are also implicated, with lower placental HLA-G expression and soluble HLA-G levels observed in PE [109]. Additionally, genetic variants of HLA-F and HLA-G show mixed associations with PE across studies [110,111,112].

HLA genotyping in PE has primarily been regarded as a research tool rather than a diagnostic tool. However, the straightforward measurement of blood anti-HLA antibodies, typically employed in transplantation and infrequently used in studies of recurrent pregnancy loss and implantation failure, warrants further investigation in the context of PE, as suggested by Lee et al. [113]. Thus, further studies on the mechanisms involving genetics, the immune response, and circulating biomarkers are required in PE.

### 7.2. Role of Killer Immunoglobulin-like Receptors (KIR) in Preeclampsia

KIRs are found mainly on NK cells and can either inhibit or activate them. It has been suggested that patients with PE have a higher presence of activating receptors [114]. Mothers lacking most or all activating KIRs (AA genotype) carrying a fetus with HLA-C2 are at a significantly increased risk of PE [115]. However, the Danish cohort showed no significant difference in maternal KIR AA frequency and fetal HLA-C2 [116]. The complexity of KIR expression in cells does not necessarily align with the observed genetic arrays, as receptor expression also depends on the tissue milieu [45].

To elucidate the role of KIR in PE, it is essential to conduct a localized assessment of KIR expression in decidual NK and T cells. Vinnars et al. [117] have demonstrated an elevated expression of NKG2D (CD314) in decidual NK cells. This heightened expression may correlate with an increased fetal expression of stress ligands for the NKG2D receptor, specifically the MICA/B and ULBP proteins. Consequently, a well-defined hypothesis warranting testing and validation should be established. Furthermore, this mechanism may contribute to the understanding of other non-classical events associated with PE.

**Figure 3:**
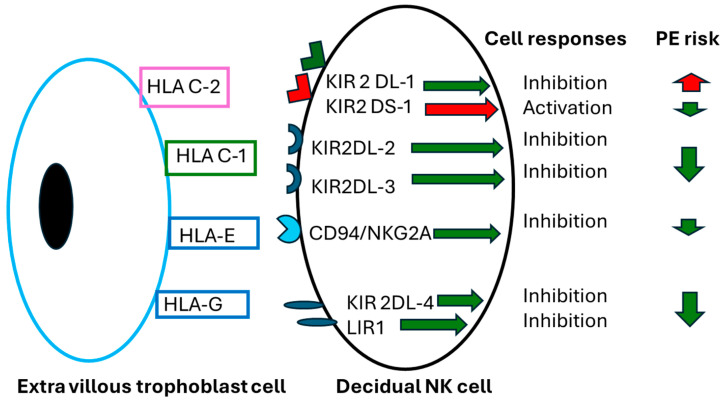
Interactions between trophoblasts and NK cells. The interactions between the HLA expressed by the trophoblast and the counterpart receptors on NK cells. HLA is the key protein expressed on the trophoblast and is dependent on the father’s genetic background. In the case of HLA-C*02, two distinct receptors on maternal NK cells are involved. The activating receptor, KIR2 DS1, activates the trophoblast, whereas the counterpart, KIR2 DL1, inhibits trophoblast function and is consequently associated with PE. In the case of HLAC1 expression by the trophoblast, the two receptors (KIR2DL2 and KIR2DL3) are inhibitory, induce a tolerogenic response in NK cells, and protect trophoblast functions. HLA-E binds to the inhibitory complex CD94/NKG2A and to HLA-G, to KIR2Dl4 and LIR1. HLA-E and HLA-G regulate inflammatory responses. A decrease in HLA-E and HLA-G expression predisposes the trophoblast to NK cytotoxic lysis upon activation.

Figure 3 illustrates the importance of HLA C, E and G in the interaction between trophoblasts and NK cells.

## 8. Immune Checkpoints in Preeclampsia

Immune checkpoints regulate immune responses and maintain tolerance [118]. PD-1 is present on most decidual cell types, while PD-L1 is found on extravillous trophoblast, syncytiotrophoblast, and maternal-fetal interface immune cells [119]. In preeclamptic placentas, PD-1 and PD-L1 levels decrease, indicating a loss of fetal immune tolerance linked to JAK2/STAT5 pathways and reduced GM-CSF regulation [119,120]. The PD-1/PD-L1 pathway affects decidual macrophage polarization, with checkpoint inhibitors potentially shifting these macrophages to M1, enhancing local inflammation [119,120]. The use of checkpoint inhibitors in therapy remains experimental, making it essential to conduct well-designed studies to evaluate their therapeutic effects.

CTLA-4 inhibits CD28-mediated stimulation of T lymphocytes and exhibits altered expression in PE [121]. The CTLA-4 49A-G polymorphism (rs231775) has been linked to reduced CTLA-4 levels, suggesting it may be a risk factor for the development of PE, although meta-analyses have yielded inconsistent findings [122]. Additionally, TIM-3 and its ligand, Galectin-9, which suppress T lymphocyte activity [121,123], exhibit altered expression in placental macrophages and Hofbauer cells in PE, potentially associated with inadequate fetal implantation [121,123,124].

The roles of various immune checkpoints, including CD276, LAG-3, and CD73, remain subjects of ongoing research. Further investigation of checkpoint control is essential to enhance our understanding of PE. However, there seems to be no direct role of checkpoint inhibitors as a therapeutic tool in PE.

## 9. Extracellular Vesicles in Preeclampsia

Extracellular vesicles, including exosomes, microparticles, migrasomes, and apoptotic bodies, are integral components of intercellular communication [125,126]. These membrane-bound vesicles serve as essential mediators of cell–cell signaling [125]. Furthermore, extracellular vesicles are posited to significantly influence and regulate placental–maternal vascular communication. In normal pregnancy, their effects can be immunosuppressive or immunostimulatory based on their origin and content [127,128]. In PE, plasma levels of placenta-derived vesicles are elevated, carrying pro-inflammatory cytokines and angiogenic factors that exacerbate vascular dysfunction and oxidative stress [129,130]. These vesicles, along with neutrophil extracellular traps, significantly damage vascular endothelia [130]. Modulating vesicle secretion is a promising therapeutic approach, and acetylsalicylic acid has been shown to reduce its harmful effects in PE [131,132,133,134].

**Figure 4:**
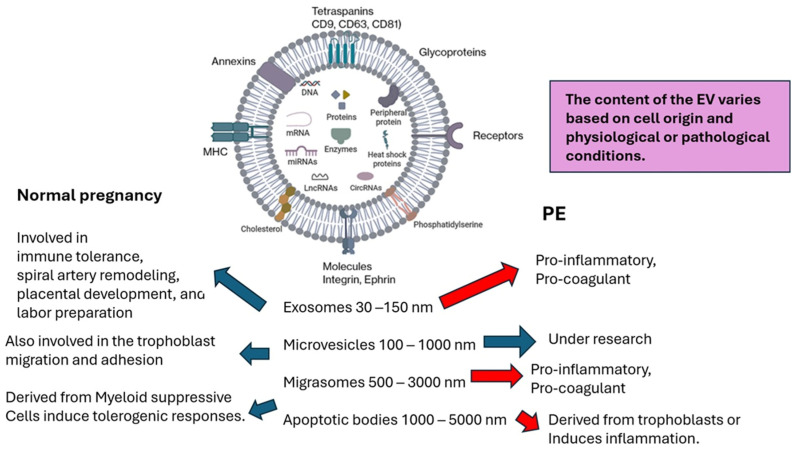
Extracellular in normal and PE pregnancies. The figure illustrates the various types of extracellular vesicles and their correlation with size and cellular processes. Centrally, the structure encompasses pro-tolerogenic or pro-inflammatory mediators, contingent upon the specific cell type and conditions. These vesicles may be characterized by the presence of tetraspanin antigens such as CD9, CD63, and CD81, as well as annexins and other mediators, as detailed within the text. It is essential to highlight the three categories of RNA: messenger RNA, microRNA, and long non-coding RNA, all of which significantly influence the target tissue. The central section of the figure is derived from a template obtained from the BioRender website (Biorender.com). The template does not include the corona complex. The content of the complex is described in the text.

The surface markers of extracellular vesicles include tetraspanins (CD9, CD63, CD81), endosomal/MVB proteins, cell-specific markers depending on vesicle origin, and other functional proteins such as annexins and heat shock proteins (Figure 4) [125,126]. However, there is growing recognition in extracellular vesicle research of the significance of the functional plasma protein corona that forms on the surfaces of lipid particles [126]. The corona is formed by apolipoproteins A1, B, C3, and E; complement factors C3 and C4B; the alpha chain of fibrinogen; and the heavy chains of immunoglobulins. This corona is known to either enhance or inhibit the uptake of extracellular vesicles, thereby influencing their size and, consequently, their functionality [126]. The impact of pregnancy and PE on the composition of the corona surrounding extracellular vesicles remains largely unexplored; therefore, further investigation is imperative to elucidate these effects.

Extracellular vesicles are crucial for a healthy pregnancy, facilitating vasodilation and modulating maternal immune responses [125,126,127,128,129]. They are released from extravillous trophoblasts, aiding in early spiral artery remodeling and placental establishment [126,127,128,129]. Previous studies show that placental-derived extracellular vesicles enhance endothelial cell proliferation and migration, as well as vascular smooth muscle cell migration during placentation. Additionally, their numbers increase in maternal circulation as gestation progresses, highlighting their importance throughout pregnancy. However, most of the authors have focused on the role of these vesicles in maternal responses rather than in local responses. Figure 4 illustrates the differences between the vesicles in normal pregnancy and PE.

In early-onset PE, the contents of vesicles appear to be associated with various biological pathways. These pathways include innate immune mechanisms and various enzymatic activities, such as catalytic and peroxidase functions [126,127,128]. Additionally, key proteins of interest include annexins, integrins, histones, heat shock proteins, and cytoskeletal proteins. (Figure 4). Nevertheless, there remains ongoing debate regarding the specificity of these proteins in relation to preeclampsia, both in early and late stages of the condition.

Hypoxia seems to enhance the biochemical composition and increase the number of placental extracellular vesicles released [126,127]. However, specific details of the contents remain the subject of ongoing research. Since both hypoxia and hyperoxia may contribute to the development of preeclampsia, these placental-derived extracellular vesicles likely play a role in the condition’s pathophysiology, particularly in damaging maternal vasculature [126,127]. Current research on the impact of extracellular vesicles in preeclampsia suggests they contribute to blood vessel injury by promoting platelet aggregation and adhesion to the endothelium. Additionally, these vesicles mediate the vascular dysfunction associated with preeclampsia, including reduced vasodilation, increased vascular inflammation, and altered cell proliferation and remodeling that are required for vascular repair.

Research on placental-derived extracellular vesicles in preeclampsia has been limited. Studies show that these vesicles can disrupt bradykinin-induced relaxation in uterine arteries, suggesting a role in vascular constriction [126,127]. However, they also reduce constrictor responses to phenylephrine and serotonin in omental arteries. In contrast, vesicles from normotensive pregnancies enhance vasoconstriction via endothelin-1 and decrease constriction from angiotensin II in subcutaneous arteries [126,127]. The exact role of these vesicles in vascular dysfunction in preeclampsia remains unclear; further investigation is needed, particularly between early- and late-onset preeclampsia, as well as in therapeutic contexts using cell lines.

Mesenchymal stromal extracellular vesicles interact with various immune components, including T and B lymphocytes, macrophages, and natural killer cells. They are crucial for reducing inflammation, modulating immune cell activation, and lowering pro-inflammatory cytokine production. These vesicles, particularly from mesenchymal stem cells, also promote angiogenesis, reduce fibrosis, and enhance cardiac function.

## 10. MicroRNAs (miRNAs) and Preeclampsia

miRNAs are small non-coding RNA molecules that regulate gene expression [135] and are linked to PE and preterm birth, potentially serving as biomarkers for these conditions [136]. Several miRs have been shown to be over- or underexpressed in PE, as illustrated in the Table 2. Notably, miR-210, which is elevated under hypoxic conditions, is present at higher levels in preeclamptic placentas and serum, whereas miR-483-3p, associated with trophoblast invasion, is reduced. Additionally [137,138], miR-155 is overexpressed in women with PE, facilitating the release of TNF-α and IL-6 [136,137,138,139]. The levels of miRs can be modulated by acetylsalicylic acid in patients at risk of PE [137].

As illustrated in Figure 4, the content of extracellular vesicles is dependent on the process, normal pregnancy, tolerogenic, or PE, proinflammatory. Although it has been suggested that miRs can be used for diagnosis or as predictive markers, standardized protocols and large clinical trials are needed to validate their use in PE. In addition, several miRs have been proposed for therapeutic purposes, and promising pharmacological tools to deliver the miRs have already been developed.

**Table 2:** miRNAs involved in PE.

miRNA Increased	Target	Observation
miR-155	(1) Alters SMAD2 and SMAD5 members of the TGFβ signaling pathway. (2) Decrease T regulatory cells, downregulate CTLA4 transcription and function. (3) Regulates Vascular Peroxidase 1 and oxidative stress and mitochondrial glucose metabolism via skeletal muscle.	Cysteine-rich angiogenic inducer 61 (CYR61)/miR-155 Ratio has been proposed as a biomarker for Diagnosis and severity of PE
miR-125b	Downregulates the Kv1.1 voltage-gated potassium channel and glypican 1, a cell surface heparan sulfate proteoglycan. It inhibits cytotrophoblast invasion and disrupts endothelial cell function.	Predictive marker and a potential therapeutic target.
miR-181a-5p	Downregulates genes involved in the MAPK/ERK signal pathway.	Associated with the worst outcomes in patients with E.
miR-29b	It inhibits Vascular Endothelial Growth Factor A (VEGFA) and reduces anti-apoptotic proteins like Myeloid Cell Leukemia Sequence 1 (MCL1), while downregulating essential enzymes (MMP2 and MMP9) required for placental cell invasion of the uterine wall.	Marker of PE severity
miR-206	Regulates genes in Muscle Development and Regeneration, tumor suppression, proliferation, neurodevelopment, and glucose-6-phosphate dehydrogenase.	Proposed as a diagnostic marker
miR-495	Impedes normal placental development by repressing Histone deacetylase 2 (HDAC2), accelerating cell proliferation, invasion, and migration, while decreasing apoptosis via the P53/PUMA pathway.	Proposed as a diagnostic marker
miR-125b	It inhibits trophoblast Invasion and function. Placental exosomes transfer miR-125b into endothelial cells, disrupting the endothelial barrier. It induces the secretion of pro-inflammatory cytokines.	Proposed as a diagnostic marker
miR-206	Inhibits trophoblast invasion and promotes placental inflammation. Suppresses the AGTR1 (Angiotensin II Receptor Type 1) gene. Downregulates VEGFA,	Diagnostic biomarker
MiR-517 and miR-526	Trophoblast dysfunction, hypoxia, and angiogenic imbalance. Endothelial damage.	Promising diagnostic marker of PE
miR-17	Suppresses key angiogenesis genes (like VEGFA, Hypoxia-inducing factor 1A (HIF1A), and the Ephrin system).	Proposed as a biomarker.
miRNA decreased	The decrease impairs	Observation
miR-21	(1) Trophoblast invasion and function are decreased, and apoptosis is increased, (2) poor spiral artery remodeling and chronic hypoxia	Lower miR-21 levels in the first trimester are associated with the future onset and severity of preeclampsia and serve as a marker for early detection.
miR-146a	(1) Proliferation, reduced invasion, and increased apoptosis of trophoblast cells, (2) Decreased spiral artery remodeling, and (3) overactivation of NFkB inflammatory pathways.	Proposed as a diagnostic marker and for PE therapeutics.
miR-126	(1) Angiogenesis, decreasing placental vasculogenesis, (2) trophoblast invasion and placental development, and (3) endothelial and trophoblast viability.	Proposed as a diagnostic marker and for PE therapeutics.
miR-195	(1) The healthy invasion and migration of trophoblast cells, (2) reduces VEGF production and angiogenesis, and (3) increases the hypoxia-induced damage and oxidative stress.	Proposed as a diagnostic marker and for PE therapeutics.
miR-363	(1) Poor placentation, including inadequate trophoblast migration and impaired spiral artery remodeling. (2) Decrease VEGFA secretion.	Proposed as an early-stage marker of PE.

Table legend: The table represents the different miRs that have been reported and proposed as good biomarkers for PE. The table was adapted from recent reviews of the literature [140,141,142].

## 11. Cytokines and Leptin in Preeclampsia

**Figure 5:**
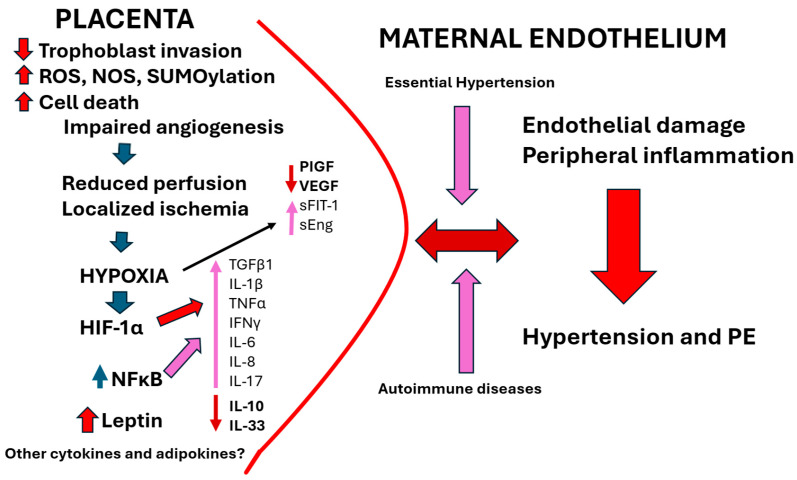
Cytokines and PE. The figure delineates the effects of various processes involved in PE and cytokine transcription and secretion. The pathophysiology of cell damage in the placenta involves elevated levels of inflammatory cytokines and mediators that affect the maternal endothelium. This induces peripheral inflammation, increases blood pressure, and activates a multi-organ response. Furthermore, individuals with a history of autoimmune diseases or essential hypertension prior to pregnancy are at an elevated risk of developing PE. The critical transcription factors in PE are Hypoxia-Inducible Factor-1 alpha (HIF-1α) and NFκB. References [143,144,145,146,147,148,149,150] were used to generate the figure.

In PE, the imbalance between pro-inflammatory and anti-inflammatory cytokines induces vascular damage and increases vascular resistance in the placental bed [143,144,145,146,147,148,149,150]. Figure 5 illustrates the cytokines involved in PE. The key tolerogenic cytokines, along with the critical growth factors crucial for vascularization, trophoblast invasion, and survival, are decreased, while the proinflammatory and activating cytokines are increased. Proinflammatory cytokines are also increased in the mother’s bloodstream. Essential hypertension and autoimmune disorders also play an important role in the proinflammatory event. These events may be exacerbated in metabolic disorders (e.g., obesity, diabetes). Still, well-designed clinical studies are required to determine the importance of genetic polymorphisms in cytokines in PE.

Cytokine gene polymorphisms such as the IL-4 VNTR [151] and the IL-17A SNP (rs2275913) have been associated with PE [152]. Gene polymorphism may aid in the generation of genetic clusters of the disease.

Leptin, a hormone produced by fat cells, is elevated in maternal circulation and placental tissue in PE and can precede symptoms such as hypertension and proteinuria, making it a potential early biomarker [153,154,155,156,157,158]. High leptin levels are associated with systemic inflammation and oxidative stress, thereby affecting maternal blood vessel integrity [149,150,151]. Our group found differences in leptin levels between mild and severe PE and noted elevated levels in hypertensive pregnant women before conception [154]. We also established a correlation between serum leptin and platelet count in preeclamptic patients, with levels decreasing after antihypertensive treatment [154]. Excess leptin is associated with vascular constriction, placental hypoxia, and increased pro-inflammatory markers [155,156,157,158]. Obesity increases the risk of PE, likely due to elevated leptin levels. Monitoring leptin in early pregnancy could help identify women at higher risk of PE [154,155,156,157,158,159,160], underscoring the urgent need for pharmacological modulation of leptin.

Further research is needed on underinvestigated cytokines with altered profiles in PE, such as IL-1β, IL-1RA [161], IL-2 [162], IL-23 [163], IL-27 [164], and IL-37/IL-38 [165].

## 12. Prostaglandins, Leukotrienes, Thromboxanes, and Resolvins

Metabolites derived from arachidonic acid, leukotrienes, and thromboxanes play a critical role in PE [166,167]. Increased thromboxane synthesis in platelets contributes to vascular resistance and platelet aggregation, which are characteristic of this disease [167]. Leukotriene B4 is elevated in serum and placentas [168], while vasodilatory prostacyclin [166] and PGE2 are notably reduced [169,170]. Although aspirin limits cyclooxygenase activity and can prevent PE, drug resistance has been described [171].

Specialized pro-resolving mediators (SPMs), such as resolvins, help resolve inflammation and prevent tissue damage by inhibiting leukocyte migration, enhancing macrophage phagocytosis of dying leukocytes, and boosting IL-10 levels [172,173]. The reports on resolving levels in PE are contradictory. Some find reduced resolvin D1 concentration in preeclamptic patients, along with a decreased resolving/leukotriene B4 ratio [174]. Other studies have demonstrated increased resolvins in these patients, particularly in those with metabolic syndrome and in early pregnancy stages [175,176]. Methodological differences account for these discrepancies, highlighting the need for structured SPM analyses alongside w-3 fatty acid supplementation trials.

**Figure 6:**
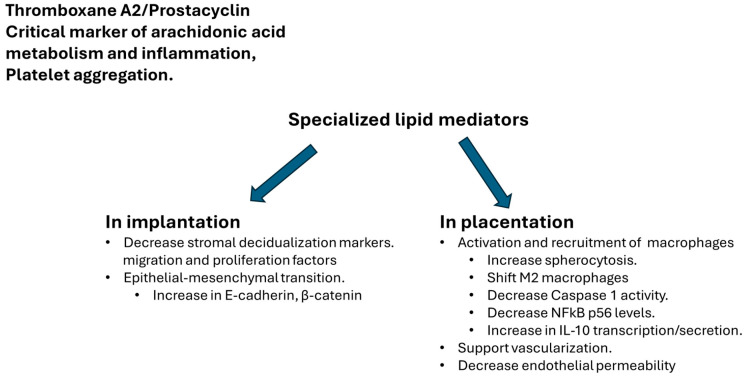
The role of SPMs in normal pregnancy, along with the contrasting effect of thromboxane A2. The effects of SPMs on implantation and placentation are illustrated, as are the opposing effects of thromboxane A2, a product of arachidonic acid metabolism. SPMs supplementation could be important in the early phases of implantation and pregnancy in women at high risk of developing PE.

Figure 6 depicts the relationship of SPMs in normal pregnancy and the influence of pro-inflammatory metabolites derived from arachidonic acid. A critical observation is that these compounds are transient and produced locally. Their biological effects depend on the expression of specific receptors, which may include non-specific receptors for these intermediates.

## 13. Radicals and Preeclampsia

### 13.1. Oxygen Radicals

The interplay between oxidative stress and PE follows a two-stage model [177]. Stage 1 involves placental ischemia, fluctuating oxygen levels, and excess ROS production due to incomplete arterial remodeling. Stage 2 sees ROS enter the maternal bloodstream, causing inflammation and endothelial dysfunction, which manifests as hypertension and proteinuria. Mitochondrial dysfunction in the placenta generates significant ROS [178] and leaked free fetal hemoglobin due to oxidative damage, which interacts with nitric oxide to generate free radicals, driving hypertension and renal impairment [179]. Additionally, up-regulated NADPH oxidase and xanthine oxidase in preeclamptic placentas further increase superoxide and free radical production [180].

Critical markers of oxidative damage in PE include malondialdehyde [181], 8-hydroxy-2’-deoxyguanosine [177], ischemia-modified albumin [182], elevated uric acid [183], and hypoxia [184]. This ROS overproduction impairs trophoblast proliferation and reduces VEGF expression [185]. While general antioxidants (vitamins C and E) lack consistent efficacy in preventing PE, mitochondria-targeted antioxidant therapies show promise [177,184,186,187]. Radical-induced senescence is also an emerging topic of interest [188].

The regulation of local production of oxygen and hydroxide radicals (as illustrated in Figure 4) is a crucial aspect of PE pathophysiology, as elevated levels of these radicals can generate various intermediates. This process subsequently induces hypoxia, cellular apoptosis, and inflammatory responses.

### 13.2. Nitric Oxide, Peroxynitrite, and Protein Modifications

Nitric oxide (NO) mediates vasodilation and regulates placental blood flow, cytotrophoblast endovascular invasion, and angiogenesis [189,190,191,192]. Inhibiting NO synthase (NOS) induces PE-like symptoms in animal models [193]. In human PE, serum NO levels (determined by its oxidation products, nitrites and nitrates) are reduced [194,195,196], leading to vasoconstriction, abnormal perfusion, and heightened pressor sensitivity [195,196,197]. The VEGF promotes vasodilation via endothelial NOS (eNOS) [198], and the combination of eNOS deficiency and sFlt-1 overexpression synergistically worsens renal dysfunction [199,200]. A connection between sEng and NO has been established [201,202], and TGF-β1, a target of sEng, initiates eNOS-mediated vasorelaxation [94]. Understanding NO metabolism is crucial for elucidating the role of the endothelium in the pathogenesis of PE [203].

**Figure 7:**
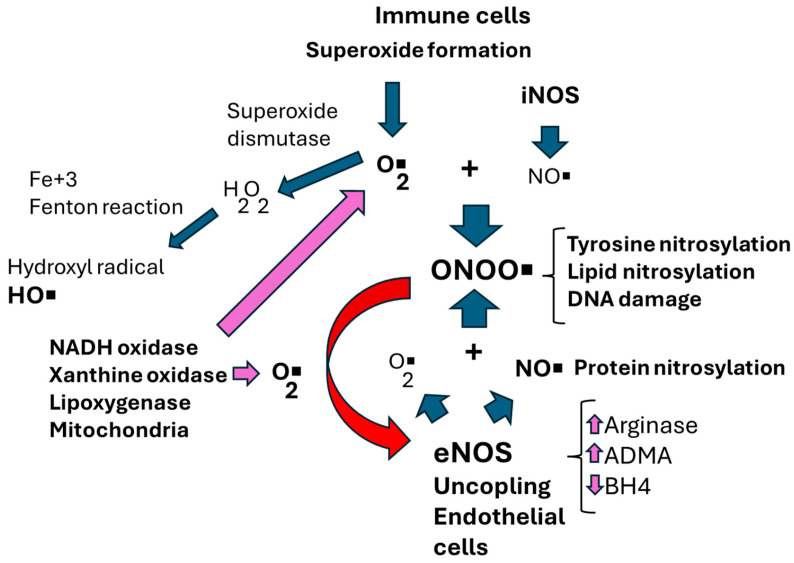
Formation of oxygen, hydroxyl, and nitrogen radicals and the effect on protein and lipid nitrosylation and DNA damage in PE. The key cellular enzymes that generate superoxide radical O_2_^●^ are NAPH oxidase, xanthine oxidase, and lipoxygenase. The increase in superoxide produced by damaged mitochondria reflects impaired function of superoxide dismutase and catalase. In the presence of free Fe3+, the Fenton reaction generates the highly reactive hydroxyl radical, which can also react with proteins, lipids, and DNA. The uncoupled endothelial nitric oxide synthase (eNOS) can also produce the O_2_● and the NO● radicals, which form the peroxynitrite radical (ONOO●). The enzyme competes with arginase for the substrate in the absence of the cofactor Tetrahydrobiopterin (BHT4) and is unable to metabolize the Asymmetric dimethylarginine dimer (ADMA). Moreover, the excess formation of NO by immune cells via inducible Nitric Oxide Synthase (iNOS) can increase NO● levels, which, in the presence of overproduced O2●, can also lead to the formation of peroxynitrite. The red line corresponds to the uncoupling effect of peroxynitrite on eNOS. The SUMOylation process was not depicted in the Figure.

When NO reacts with superoxide, it forms peroxynitrite, a potent oxidant implicated in endothelial dysfunction in PE. This results in protein nitration and higher levels of 3-nitrotyrosine, a marker for PE [182,191,192,193]. Elevated peroxynitrite levels in maternal blood and placenta lead to oxidative stress, increased vascular resistance, and decreased NO availability [195,204,205,206]. Peroxynitrite activates the NFκB pathway, resulting in enhanced expression of the lectin-type oxidized LDL receptor (LOX-1) and increasing the transcription of arginase II. Increased Arginase II depletes cellular L-arginine, an essential substrate for eNOS (endothelial nitric oxide synthase). This leads to eNOS “uncoupling,” resulting in increased superoxide production rather than the protective NO, which worsens oxidative damage [195,207]. Barbosa et al. reviewed the potential role of oxidative stress modulation in PE [203]. The events are summarized in Figure 7.

**Figure 8:**
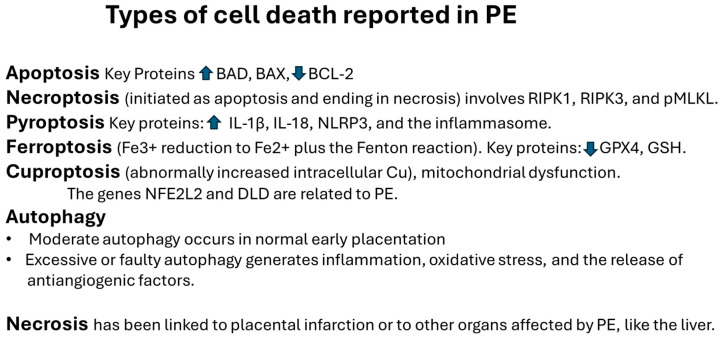
Types of cell death reported in PE. The key elements related to each type of cell death are described. The figure is based on references [216,217,218,219,220,221,222,223,224,225,226,227,228,229,230].

Excessive NO radicals lead to S-nitrosylation of proteins, hindering placentation [208] and promoting cell death [209]. Kulandavelu et al. [210] suggested that increased S-nitrosylation in placentas results from a deficiency of S-nitrosoglutathione reductase associated with preeclampsia. This enzyme deficiency has also been associated with various pathologies, including diabetes, through its effects on autophagy [211]. Figure 8 illustrates the different types of cell death in PE.

Hypoxic stress in PE also enhances protein SUMOylation, leading to trophoblast shedding and decreased PlGF [212,213]. Thereby, exosome SUMO protein analysis is a potential tool for predicting preeclampsia [214]. A low dose of aspirin may affect SUMOylation [215].

## 14. Cell Death in Preeclampsia

PE is characterized by increased trophoblast cell death due to oxidative stress, hypoxia, protein modifications [216,217,218,219,220,221,222,223,224,225,226,227,228,229,230], and nutrient deprivation [228,230]. Apoptosis is a normal process in pregnancy and is enhanced in PE [216,217]. On the other hand, necroptosis culminates in necrosis after inefficient apoptosis, releasing stress proteins and danger signals that amplify the inflammatory response [219,220,230]. Pyroptosis is also an interesting phenomenon in PE, probably linked to the genetic proinflammatory background [221,222]. Ferroptosis is linked to a complex process involving reactive radicals [223,224,225]. This cell death releases syncytial knots and debris into the maternal circulation, triggering systemic inflammation and endothelial dysfunction through danger signals that activate the NLRP3 inflammasome pathway [228]. Cuproptosis has been reported in PE and its genetic link to possible predisposition [226,227].

Figure 8 shows the different types of cell death reported in PE, along with the major intermediates. It is important to stress that apoptosis, necroptosis, autophagy, pyroptosis, ferroptosis, and cuproptosis have been reported in the placenta, whereas necrosis [218] has been reported in maternal liver as a consequence of severe PE.

## 15. Infectious Diseases and Preeclampsia

Bacterial, viral, and parasitic infections trigger inflammatory cascades pivotal to PE pathogenesis [231,232,233]. The TORCH complex (Toxoplasmosis, Syphilis, Rubella, cytomegalovirus, Herpes/HIV) and, more recently, the Zika virus, are responsible for transplacental transmission and complications [233,234,235,236,237]. Other pathogens, including *Chlamydia trachomatis* [238], *Toxoplasma gondii* [239,240,241], *Trichomonas vaginalis* (which increases IL-8 and galectin secretion) [242], *Plasmodium* and *Trypanosoma cruzi* [243,244], represent elevated PE risks.

Malaria, particularly Plasmodium falciparum, is a major risk factor, leading to decreased uterine perfusion, reduced NO production, and increased levels of sFlt-1, endoglin, and vascular endothelial growth factor receptor 1 (a known PE marker) [245,246,247,248,249]. In patients with malaria-preeclampsia comorbidity, placental villous maturity and villous volume density are significantly reduced in comparison with normal pregnancy [249].

Viral infections such as cytomegalovirus, adeno-associated virus 2 (AAV-2), SARS-CoV-2, herpes simplex virus, Epstein–Barr virus, and HIV are associated with an increased probability of PE [250]. Von Dadelszen et al. [250] reported elevated anti-CMV antibody levels in patients with early-onset preeclampsia. Moreover, increased Toll-like receptor 2 and 4 expressions have been reported in CMV infection and preeclampsia [251,252], accompanied by elevated IL-6 and TNF-α levels and reduced IL-10 [251,252]. Placental AAV-2 infection rates were significantly higher in patients with preeclampsia than in normotensive patients [253], and a 5.6-fold increase in anti-AAV-2 IgM was observed in preeclampsia patients who experienced fetal growth restriction, preterm birth, or fetal demise [254]. SARS-CoV-2 induces systemic endothelial damage and microvascular dysfunction mimicking PE, though definitive diagnostic criteria suggest the virus exacerbates comorbidities rather than directly increasing PE incidence [255,256,257,258,259,260,261,262].

**Figure 9:**
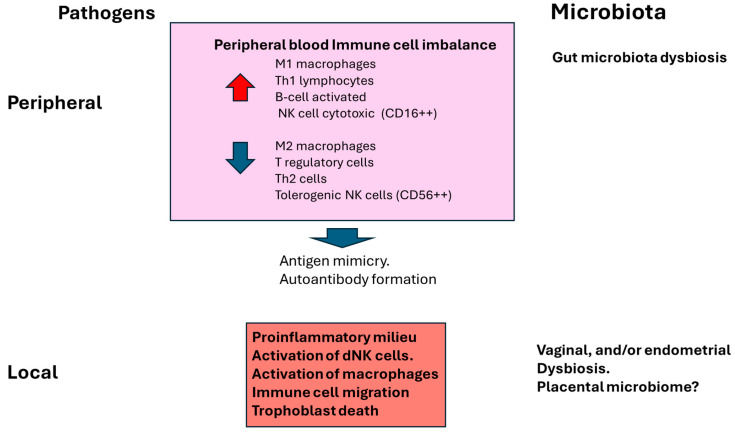
The impact of infection and microbiota dysbiosis on both peripheral and localized immune responses is significant. Infections caused by pathogens, as well as dysbiosis of the gut microbiota, can activate the peripheral immune response, subsequently influencing the interactions between immune cells and trophoblast function at the local level. Furthermore, these conditions can trigger autoimmune responses through mechanisms such as antigen mimicry. Localized infections within the uterus can alter the decidual environment, provoking a local inflammatory response that may result in the apoptosis of trophoblast cells. This information is derived from existing literature on the subject [231,232,233,234,235,236,237,238,239,240,241,242,243,244,245,246,247,248,249,250,251,252,253,254,255,256,257,258,259,260,261,262,263,264,265,266,267,268,269,270,271]. The possible role of the placental microbiome is still controversial, and it is highlighted by the question mark.

In summary, inflammatory responses to infections play a pivotal role in the development of PE (shifts from Th2 to Th1 cytokine profiles, elevated pro-inflammatory cytokines, increased oxidative stress, and upregulated anti-angiogenic proteins (Figure 9) [232,233]. More research is needed in this area.

## 16. Microbiota and Preeclampsia

Recent evidence shows distinct microbiota in the uterine cavity, vagina, and placenta, challenging the “sterile womb” hypothesis [263,264,265]. Dysbiosis during pregnancy affects metabolic and immune homeostasis, potentially contributing to PE (Figure 9) [266,267,268,269,270,271,272,273,274,275,276,277,278]. Third-trimester microbiota from women with PE resemble those associated with metabolic diseases, and fecal transplants from women in the third trimester cause weight gain and inflammation in mice [274,275,276,277,278]. In PE, beneficial bacteria such as *Lactobacillus* decrease, while *Saccharibacteria* increase [277,278]. Fecal transplants from patients with PE trigger inflammatory responses in pregnant rats, suggesting that probiotics or fecal transplants may be potential therapies [267,271].

## 17. Autoimmune Diseases and Preeclampsia

Rheumatic autoimmune diseases have an 8–10% prevalence and notably increase the risk of PE, infertility, and intrauterine growth restriction (25.3% vs. 6.1% in healthy controls) [279,280]. Antiphospholipid Syndrome (APS) is characterized by the presence of antiphospholipid antibodies, thrombotic events, and/or obstetric complications, which can result in inadequate trophoblastic invasion [281,282,283,284]. Between 25% and 50% of women with APS experience PE, and antibody levels correlate with disease severity [281,282,283,284,285,286]. Similarly, patients with Systemic Lupus Erythematosus (SLE) have a threefold risk of PE [286,287,288,289,290,291]. A recent study in Sweden demonstrated a reduction in the proportion of patients with SLE who had PE over the past two decades, likely associated with the use of hydroxychloroquine and low-dose aspirin [292].

Although pain in ankylosing spondylitis (AS) often improves in early pregnancy [293], there is an increased risk of fetal growth restriction [294]. One study demonstrated an increased risk of PE in patients with psoriatic arthritis but not in pregnant patients with AS [295].

Women with systemic sclerosis have a higher incidence of PE [296,297,298]. Conversely, having a history of PE increases the subsequent risk of developing scleroderma by 69% [298]. A case–control study found that women who later developed scleroderma also had a higher incidence of hypertension during pregnancy [299].

Both hypothyroidism and hyperthyroidism increase the risk of PE [300,301,302]. A meta-analysis found that patients with PE often had abnormal thyroid function test results [303]. One study indicated that autoimmune thyroiditis slightly increased the risk of PE [304], whereas another did not find a link between thyroid autoimmunity and PE [305].

There are still many unanswered questions relating to autoimmunity and PE. However, autoantibodies can be a useful marker for clinical management during pregnancy (Table 3).

## 18. Atopic Dermatitis (AD) and Preeclampsia

AD is linked to a modestly increased risk of PE, and PE is a prenatal risk factor for childhood eczema, suggesting a bidirectional relationship between immune programming in PE pregnancies and the atopic spectrum. This correlation may involve immune mechanisms, including NK cell pathways influenced by KIR genes [310,311,312]. These findings have opened new avenues for research and necessitate well-structured clinical trials to determine the relationship between allergic diseases and PE.

## 19. Immunological Treatments

Current therapies for PE emphasize prevention in high-risk women. Currently, apart from childbirth, there are no approved treatments that can effectively halt the progression of PE once it has been established. A summary of various published treatments [313,314,315,316,317,318,319,320,321,322,323,324,325] is provided below.

**Figure 10:**
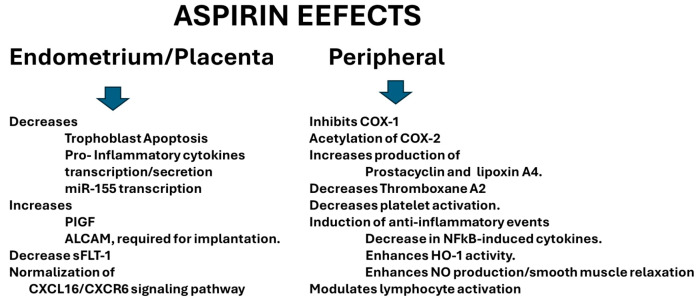
Effects of aspirin on endometrial, placental, and peripheral tissues. The effects of aspirin depicted in the figure were obtained from references [313,314,315,316,317,318,319,320].

Low-dose aspirin (LDA) remains the first-line preventive treatment, reducing the risk of PE by 62% through anti-inflammatory and pro-angiogenic effects [313,314,315,316,317,318,319,320]. Figure 10 illustrates the effects of aspirin treatment on the endometrium/placenta and peripheral. The effect of low-dose aspirin can be considered protective in patients at risk. Nonetheless, large-scale clinical trials may be important to ascertain the effect of the therapy at early stages and during pregnancy as a monotherapy or with heparin.

**Figure 11:**
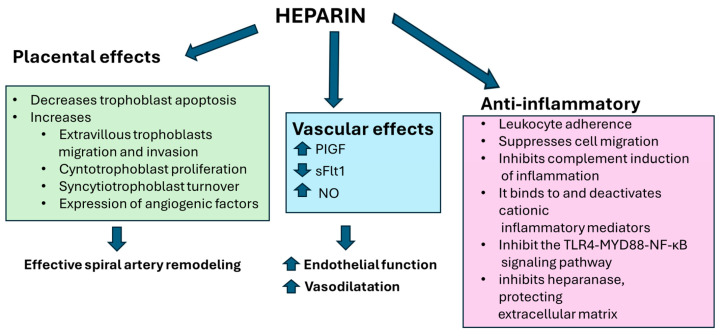
Proposed local, vascular, and anti-inflammatory effects of heparin in PE. The effect of heparin has been depicted from [314,320,321,322,323,324,325].

Low molecular weight heparin has been proposed as an effective treatment for high-risk PE patients [321,322,323,324,325]. According to a meta-analysis, the combination of LDA and low-molecular-weight heparin significantly reduces the risk of PE in patients with APS [314]. The protective effects of heparin have been summarized in Figure 11.

**Figure 12:**
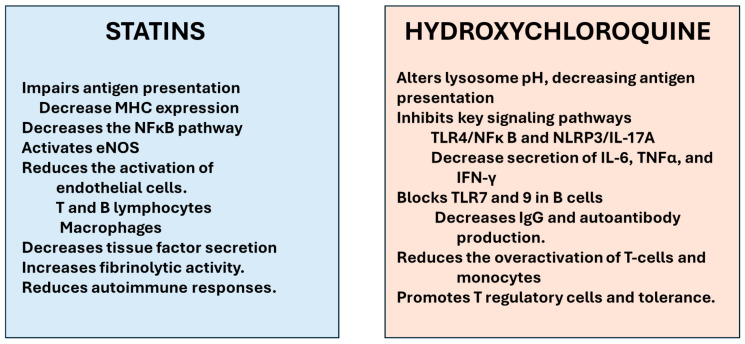
Comparison of the proposed effects of statins and hydroxychloroquine (HCQ) on immune response modulation in PE. References [326,327,328,329,330,331,332] were used to make the figure.

Hydroxychloroquine (HCQ) has been shown in various studies to reduce the NF-κB/sFlt-1 ratio and increase PlGF levels [315,326,327,328,329]. Multicenter trials involving high-risk populations, specifically those with a history of preeclampsia and autoimmunity, indicate that HCQ may decrease the incidence of PE [315,326,327,328,329]. In Figure 12, the effects of HCQ have been compared with those of statins since both drugs decrease antigen expression.

The use of statins in PE has had controversial results [330,331,332]. Although in principle, statins could decrease the inflammatory response by reducing inflammation, antigen expression, and immune cell activation [330], meta-analysis of clinical trials has shown no conclusive effects [331]. However, pravastatin appears to correct dysfunction in the uterine radial arteries ex vivo. Figure 12 compares the effect of both treatments; nevertheless, well-designed clinical trials are required to define whether HCQ treatment is appropriate in patients without autoimmune disease and whether statin therapy can be an adjunct therapy for PE patients.

Regulatory dendritic cells (DCregs) play a pivotal role in modulating the immune response by facilitating the conversion of T-helper cells from Th1 to Th2 phenotypes. This shift results in an increased IL-10-to-TGF-β ratio. Preclinical studies using Hmox1^−^/^−^ mice, a model of PE, have demonstrated that administering DCregs can prevent the onset of hypertension, proteinuria, and fetal loss [333]. Moreover, mesenchymal stem cell-derived extracellular vesicles (MSC-EVs) have been shown to immunomodulate uterine NK (uNK) and myeloid cells, elevating the anti-inflammatory cytokine IL-10. In a preclinical study using the Hmox1 model, MSC-EV administration normalized placental morphology and improved fetal growth [334].

Preclinical studies suggest that anti-TNF-α therapies (etanercept, infliximab, and adalimumab) may help prevent PE and improve maternal blood pressure in certain models [335,336,337]. However, human data are limited, and these therapies have been associated with risks such as intrauterine growth restriction, spontaneous abortion, and preterm birth [338]. Notably, in patients with inflammatory bowel disease, there seems to be a trend indicating a protective effect against PE [339].

C5aR antagonists help minimize C5a-induced trophoblast injury. Eculizumab, a monoclonal antibody that inhibits C5, shows beneficial effects in PE [340,341,342,343,344]. Even though complement regulation is critical in PE, more studies are required to establish the benefits of the therapy and to compare it with lower-cost therapies that are likely to have a similar effect.

Other compounds have been analyzed in small trials; consequently, caution is warranted when interpreting those results [345]. Other therapies shown in Figure 10, mostly at the preclinical stage, focus on treating the placenta using nanopharmacology.

Recently, it has been proposed that heterogeneity in pharmacological responses may also be influenced by distinct patient subgroups. Variations in genetic background and the presence of hypertension prior to pregnancy may predispose individuals to different therapeutic responses [346]. The analysis of cell-free DNA may significantly aid in identifying specific patient subgroups in the early stages of PE, thereby optimizing the effectiveness of pharmacological treatments [347].

## 20. Future Perspectives

Future research directions necessitate the implementation of additional clinical trials aimed at achieving the following objectives: (1) to identify specific subclusters of patients diagnosed with preeclampsia (PE), (2) to establish effective therapeutic regimens, (3) to determine unique biomarkers indicative of PE risk and for monitoring purposes, (4) to conduct genetic analyses in women at high risk to identify potential associations, (5) to investigate the relationship between the risk of autoimmune disorders and the elevated risk of PE, (6) to evaluate whether pharmacological preventive therapies should be administered to reduce the incidence of PE among at-risk populations before conception, and (7) to assess the efficacy of antihypertensive treatment in women with hypertension to prevent superimposed PE.

**Figure 13:**
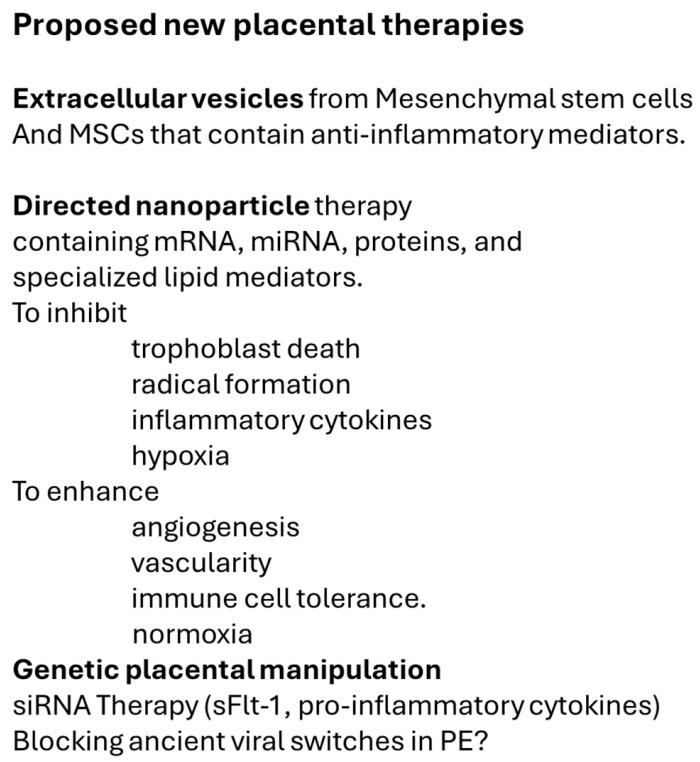
A schematic view of the proposed new placental therapies is depicted.

Various immunological therapies are being investigated to enhance prevention and treatment strategies, with a focus on personalized approaches. Nanotherapeutics also represent a significant opportunity for future therapeutic interventions [348]. The development of placental-targeted therapies is gaining momentum, with validated biomarkers driving future progress. Figure 13 illustrates the new proposed therapies.

## 21. Conclusions

From an immunological perspective, PE arises from a failure of maternal-fetal tolerance, characterized by inadequate spiral artery remodeling, elevated Th1/Th17 responses, decreased Tregs, NK cell dysfunction, M1 macrophage polarization, autoantibodies, increased oxidative response and protein modification, altered cell death, and an anti-angiogenic imbalance [10,11,12]. Dysbiosis of the microbiota, infections, hypertension, metabolic alterations, and autoimmunity increase the risk of preeclampsia through Th1 and dysbalanced cytokine pathways.

**Figure 14:**
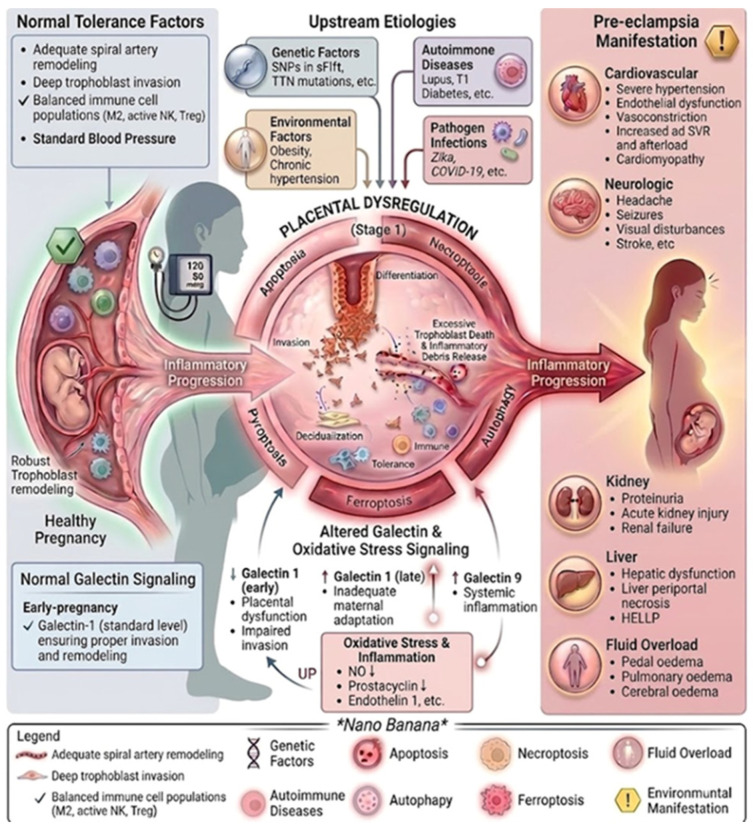
Pathogenesis of preeclampsia: from upstream etiologies to systemic manifestations. The transition from a healthy pregnancy (**left**) to PE is driven by upstream factors triggering placental dysregulation, altered galectin signaling, and oxidative stress (**center**). This local dysfunction drives systemic progression to severe maternal complications (**right**). Importantly, the immune response plays a crucial role in all these scenarios; therefore, changes in immune response are expected across these conditions and generated via Nano Banana.

In addition to the clearly defined stages of PE, some researchers have suggested that these stages be further categorized into subgroups based on factors such as age, metabolic status (including high body mass index and various endocrine alterations), genetic predisposition, and prior hypertension. Notably, the immune response is a critical factor in all of these scenarios; consequently, alterations in immune response are anticipated across these conditions. Figure 14 summarizes the points discussed in the review.

Biomarkers are essential in the early identification of diseases and in assessing the effectiveness of therapeutic interventions. Integrated biomarkers, such as sFlt-1, galectins, and PlGF, have significant potential to enable early clinical interventions. Additionally, detecting oxidative products, oxidized lipids, nitrotyrosine, and S-modified proteins may further enhance the utility of these integrated biomarkers. However, there are no clear universal guidelines. Further research is required to validate various candidate biomarkers, as they are crucial for determining treatment responses and monitoring disease progression.

The therapeutic management of PE is an area of ongoing development; however, the rising incidence has intensified the urgency to establish consensus on effective interventions. Evidence indicates that low-dose aspirin and low-molecular-weight heparin are effective in preventing PE among high-risk patients. HCQ has shown potential as a preventive measure for PE in individuals with pre-existing autoimmune disorders. Conversely, certain therapies, such as anti-TNF agents or cellular therapies, appear to be less favorable than targeted pharmacological treatments. Future therapies that combine nanotechnology and placental therapy are gaining attention, although their scope may be limited. In summary, further research is imperative on the pathophysiology and treatment of preeclampsia.

## Figures and Tables

**Table 1:** sFlt-1/PlGF ratio in predicting preeclampsia onset and associated complications [2,87,90].

sFlt-1/PlGF Ratio	Result	Interpretation
<38	Negative test.	Excludes the diagnosis of preeclampsia in patients with suspected preeclampsia. Indicates a low risk of preterm birth.
≤38 y < 84	Positive test abnormal	Suggestive of placental dysfunction. Increased risk of preterm birth.
≥85	Positive test abnormal	Indicates placental dysfunction. Effectively predicts the onset of PE, with a high risk of preterm birth and associated complications.

**Table 3:** Relevant Autoantibodies in PE.

Antibody	Pathologic Effects	Ref.
Antiphospholipid antibodies	Increased blood clotting and damage to the endothelial lining, leading to vasoconstriction and high blood pressure.	[284,290]
Anti-thyroid antibodies	Affect the function of the thyroid gland. May interact with antigens in the fetus or placenta.	[302,303,304,305]
Anti-α adrenergic receptor	It may stimulate placental α-adrenergic receptors, thereby elevating blood pressure.	[306,307]
Anti-β adrenergic receptor	The results are controversial and need to be confirmed through large-scale studies.	[306]
Anti-angiotensin II type I	The autoantibodies may cause hypertension and other abnormalities by increasing sFlt-1 and ET-1 levels.	[308]
Anti-muscarinic receptor	The results are controversial and need to be confirmed through large-scale studies.	[309]

## Data Availability

No new data were created or analyzed in this study. Data sharing is not applicable to this article.
